# “*I need to feel safe before I can engage*”: embedding trauma-informed principles in sexual and reproductive health digital technologies

**DOI:** 10.3389/fdgth.2026.1733713

**Published:** 2026-03-05

**Authors:** Agnes Kyamulabi, Abdul-Fatawu Abdulai

**Affiliations:** 1 School of Population and Public Health, University of British Columbia, Vancouver, BC, Canada; 2 School of Nursing, University of British Columbia, Vancouver, BC, Canada

**Keywords:** digital health, focus group discussions, sexual and reproductive health, technology-related trauma, trauma-informed care

## Abstract

**Background:**

The use of digital health technologies to access information and services related to sexual and reproductive health has been increasing. Despite the usefulness of these technologies, there are emerging concerns that they could inadvertently trigger, perpetuate and exacerbate trauma among patients. The purpose of this study was to explore trauma-informed care principles that could be applied in designing and/or utilizing sexual and reproductive health services.

**Method:**

We conducted 5 focus group discussions with participants who have used digital health technologies to access sexual and reproductive health services in Western Canada. The discussion centred on ways sexual health-related digital technologies could prevent triggering or perpetuating trauma among patients. The discussion took place over Zoom, and the data were analyzed using a thematic analysis approach.

**Results:**

The study revealed five main considerations that could be adopted in the design and use of sexual and reproductive health technologies to prevent the unintended consequences of trauma. These include (1) integrating accessibility and inclusivity features; (2) integrating confidentiality, safety, and privacy features like quick exit buttons; (3) using empathetic language and terminologies; (4) integrating emotional and psychological support services; and (5) implementing aesthetic design features.

**Conclusion:**

The findings of this study would help produce equitable, safe, and empowering digital health technologies for all users, particularly trauma survivors. By integrating these principles, developers and healthcare providers can create tools that reduce barriers, mitigate re-traumatization risks, and promote positive health outcomes. Future research should focus on evaluating the implementation and impact of trauma-informed digital tools in diverse settings.

## 1 Introduction

Sexual and reproductive health (SRH) services, including contraception, sexually transmitted infections (STIs) prevention and treatment, HIV/AIDS, menstrual health, and maternal health is a critical aspect of public health (1–3). The World Health Organization (WHO), through its Global Strategy, has emphasized the importance of ensuring equitable access to sexual reproductive health rights (SRHR) (2). This strategy focuses on improving family planning, eliminating unsafe abortion, addressing sexually transmitted infections, and promoting sexual health (2). Despite these efforts, accessing SRH services can be fraught with challenges, particularly for marginalized groups, including sex workers, Two-Spirit, Lesbian, Gay, Bisexual, Transgender, Queer, Intersex, and Gender-diverse (2SLGBTQI+) people, racialized communities, and survivors of intimate partner violence (4–6). These groups often face intersecting vulnerabilities, including systemic inequities, stigmatization, and limited access to social and healthcare resources (7–9). This marginalization exacerbates their risk of emotional trauma, as they navigate sensitive and stigmatized issues while contending with systemic barriers and societal judgment. For example, survivors of sexual violence or those living with HIV may encounter discrimination and breaches of confidentiality, adding to their emotional distress and creating long-term barriers to care (9, 10).

Given the challenges with accessing sexual and reproductive health services, digital health technologies have emerged as innovative tools to improve access to SRH services, offering new opportunities to address inequities (11, 12). Platforms such as menstrual tracking apps, HIV/AIDS applications, contraception and abortion web-based platforms, and telemedicine applications have enabled more inclusive and far-reaching care (13–17). These technologies are particularly valuable for individuals in underserved or stigmatized communities, providing a degree of anonymity, autonomy and flexibility that traditional healthcare settings often lack (4, 18). However, while digital health technologies have the potential to mitigate barriers to care, they also pose significant risks of re-traumatization, particularly for users with prior traumatic experiences (4, 11, 19). Re-traumatization in this study is defined as the awakening of distressing memories associated with past trauma, often triggered by experiences that resemble previous violations of privacy, confidentiality, autonomy, and safety (20). Digital technology platforms may contribute to re-traumatization through presenting ethical concerns related to data security, consent, confidentiality and privacy (21). Considering SRH, these concerns are sensitive as these technologies collect personal information and operate in environments influenced by surveillance, health inequities, stigma, and gender inequities. When ethical safeguards are weak, digital health technologies can accidentally reproduce discrimination and structural harms, reinforcing stigma and undermining users' trust in health systems. All these amplify feelings of vulnerability and distress (4, 11, 18, 22).

Re-traumatization can also arise from intentional harm, such as cyberbullying or surveillance, or unintentional consequences, such as poorly designed interfaces that expose sensitive information or evoke painful memories (11, 12, 23, 24). Research on technology-facilitated trauma highlights how digital platforms can exacerbate pre-existing traumas or create new forms of distress (4, 12, 18, 21, 25–27). For instance, period tracking apps have been criticized for reinforcing gender norms, reducing sexual privacy, and sharing users' sensitive data with third parties without proper consent (18, 25). Similarly, HIV prevention apps have raised concerns about privacy breaches and inadequate data protection, which can cause anxiety and mistrust among users, particularly those already navigating stigma or discrimination (10, 22). These ethical gaps or errors are not just technical oversights but can also lead to mental, emotional and psychological harms among people with past experiences of trauma. For marginalized populations such as survivors of sexual violence, sex workers, and 2SLGBTQI + individuals, undocumented migrants, technology can amplify existing traumas tied to their SRH experiences (6, 12, 28–32). Survivors of violence who turn to digital platforms for contraceptive services may encounter re-traumatization when algorithms present them with triggering content or inappropriate advertisements (11, 32–34). Additionally, sex workers utilizing online platforms for health information or access to services may be subjected to surveillance or targeted by cyber-harassment campaigns, compounding their trauma (29). In some cases, technologies designed for sexual and reproductive health are ill-equipped to address the unique vulnerabilities of these groups, failing to consider their histories of trauma and the cultural stigmas they navigate daily. Survivors of violence may encounter triggering content or inappropriate advertisements when using digital platforms for contraceptive services (23, 32, 35). 2SLGBTQI + individuals and sex workers who turn to online tools for health information or services may face targeted harassment, surveillance, or breaches of confidentiality (5, 29, 31, 36). These experiences not only amplify existing traumas but also undermine trust in digital health solutions, discouraging engagement and limiting the effectiveness of interventions designed to improve health outcomes (10, 22, 24, 34). They demonstrate how ethical oversights in technology design can deepen pre-existing trauma and worsen health inequities.

Despite the growing concerns of how digital health technologies can inadvertently retraumatize, there is limited guidance on how ethical and trauma-informed care can be incorporated into the design and deployment of sexual and reproductive health-related digital technologies. The purpose of this study was to explore patient-centred trauma-informed approaches to designing and deploying sexual health-related digital health technologies. This study is essential to ensuring that sexual and reproductive health-related digital technologies address the unique needs and vulnerabilities of users without triggering or perpetuating prior trauma. Secondly, by prioritizing the trauma experiences of service users, we aim to prevent technology-facilitated trauma and foster inclusive, sensitive, respectful, ethical and effective technology solutions for improving access to sexual and reproductive health services.

## 2 Methods

We conducted five virtual focus group discussions with people who use sexual and reproductive health-related technologies to gather insights on how these technologies could be designed or used to prevent the unintended effects of trauma. The focus groups comprised 4–5 participants per group. A focus group discussion was adopted because it facilitates interaction among participants, allowing them to comment, explain, challenge, and build on one another's views—insights that may not emerge during individual interviews. Because participants were not expected to share personal experiences but rather to propose solutions to technology-related trauma, this approach was considered appropriate (37, 38). In this study, technology-related trauma refers to how the use of digital health technologies and their interface features, could inadvertently trigger new forms of trauma or perpetuate and exacerbate prior trauma. Re-traumatization occurs when a digital health technology triggers prior trauma.

### 2.1 Researcher positionality and reflexivity

We are researchers originally from Sub-Saharan Africa (SSA), studying and working in a western institution. We both identify as members of racialized minority groups. These intersecting identities fostered a shared sensitivity to the inequities and traumas experienced by individuals navigating sexual and reproductive health services. We acknowledge that our work is deeply influenced by prior evidence, as well as our own experiences and perspectives. Given our background, we were more likely to have pursued a certain line of query or focus on data elements that resonated with our own personal experiences. Also, our personal experiences might have made it difficult to “step back” and analyze the data objectively, thereby leading to missed nuances. Our affiliations with Western academic institutions and health systems may carry implicit assumptions about care, technology, and expertise. To address these tensions, we engaged in ongoing reflexive dialogue throughout data analysis, questioning how our disciplinary training and lived experiences influenced interpretation. We maintained analytic memos (informal and ongoing notes written by the researchers) which were used throughout the analysis to support reflexivity and analytic rigor. During the coding process, we regularly compared emerging codes and coding categories with the analytic memos to confirm, refine, or refute interpretations of the data. These memos documented analytic decisions, reflections on patterns and relationships within the data, and points of uncertainty. These reflexive process ensured that our interpretations honoured participants' perspectives and that our analysis remained grounded in the trauma-informed principles guiding the study.

### 2.2 Context of the study

The study was conducted in the province of British Columbia (BC), located in western Canada. This setting was selected because of the widespread availability and use of digital health interventions for accessing sexual and reproductive health information and services. Given that the literature on trauma-informed design of sexual and reproductive health technologies remains limited, we aimed to generate context-specific insights that could serve as a foundation for future, larger-scale studies.

### 2.3 Participants

The population for this study includes people who have used digital health platforms to access sexual and reproductive health services. To be eligible for inclusion, participants had to: (1) have used a digital health technology (e.g., STI testing services, telehealth or video-based consultations, online booking systems, or mobile health applications) to access sexual health information, consultation, treatment, referral, or counselling services within the past year; (2) be 19 years of age or older; and (3) be sufficiently fluent in English and have access to a smartphone, computer, or laptop with a Zoom account. In this study, digital health technologies are used to refer to government and/or not-for-profit regulated web-based platforms or mobile phone applications that are used to provide sexual and reproductive health services, including contraception, abortion, STI testing, medical appointments, and teleconsultation services. We focused on government-approved technologies due to the general distrust patients may have toward privately/for-profit developed sexual and reproductive health technologies and the potential for unscrupulous actors to misuse such platforms to cause harm (21, 39).

### 2.4 Recruitment

The participants for this study were obtained through two main sources. These include recruitment through ReachBC and by contacting participants who have participated in previous studies. As a first step, we posted the recruitment message on REACH BC, a provincial web-based platform that connects volunteers and researchers within the province of BC. ReachBC is a provincial and leading health research platform in BC that connects researchers and volunteers in various health studies (40). The study poster was then circulated to potential participants who met our inclusion criteria. Participants who expressed interest were contacted by the first author and then screened to make sure that they indeed met the eligibility criteria. As a next recruitment approach, we also reached out to participants who have taken part in our prior studies and have consented to be contacted for further studies (21, 41–43). Interested participants contacted the study coordinator, and we then followed up by telephone to confirm eligibility and respond to any questions regarding study participation. Eligible participants subsequently received an email invitation to indicate their availability for the focus group discussions. Later, a consent form plus other study materials were shared with the participants, including the ground rules or group norms. Recruitment was finalized based on participants' confirmed availability, signing of consent forms and sessions were scheduled at mutually convenient times. Each participant was assigned a pseudonym that they will use when joining the Zoom channel for the focus group discussion. These pseudonyms were used to make sure that participants remain anonymous to other participants and are comfortable speaking.

### 2.5 Study procedures/data collection

Before the focus groups, participants were asked to reflect on the various technologies that they used to seek sexual and reproductive health services, information or services like STI testing and treatment, contraception (pills, IUDs, condoms, shots), abortion services, and sexual health. The second author also took field notes during the focus group discussions. The discussions centred on participants' experiences with sexual and reproductive health digital platforms and their recommendations for ensuring trauma-informed design and utilization of digital health technologies for sexual and reproductive health services. Thinking of the technologies participants have used, we specifically asked them questions like “What are your experiences in using digital technologies to access sexual and reproductive health services; How can technologies be designed to be more user friendly for people who have experiences of trauma; How should sensitive information be presented to avoid causing distress or triggering trauma, are there some specific words, phrases or images that you think should be avoided/included, while passing on this sensitive information; What privacy features are essential that can prevent feelings of vulnerability or exposure to trauma”. The focus group discussions were facilitated by the lead author, with assistance from the second author. These FGDs were conducted using the University of British Columbia's accredited Zoom platform. The discussions were audio-recorded and then transcribed verbatim for analysis. We debriefed after each FGD, a process that allowed for reflections on our interaction and early identification of key themes. Each group FGD lasted approximately 60 min.

While we did not ask participants about any traumatic experiences, we acknowledge the sensitive nature of the topic and the risk of re-traumatization. Therefore, we followed the highest ethical standards and adopted trauma-informed research approaches to ensure the privacy, safety and confidentiality of the participants before, during and after the data collection. Prior to data collection, we developed self-care resources (including contact details for counsellors) to support participants' emotional well-being during and after the FGDs. We also encouraged participants to find safe, secure and convenient places for logging into Zoom to ensure their privacy and safety. During the focus group discussion, we asked participants to turn off their video functions on Zoom, and each participant was assigned a pseudonym to ensure their anonymity. We also paid attention to the emotional state of the participants and sent them a private message to take a break if we realized any sign of discomfort in their responses. At the end of the focus group, we gave participants time to ask any questions they might have. We encouraged participants to use the self-care resources and provided referral pathways to mental health services and community organizations that can provide support. We intentionally decided not to collect some personal information including race and ethnic identities in this study as that could have affected participants' comfort, confidentiality, and willingness to engage openly. Ethical approval was obtained at the University of British Columbia Behavioural Research Ethics Board (REB# H23-03934). Each participant provided written informed consent, and each of them was provided with an Amazon gift card honorarium worth CAD30 (USD22) at the end of the focus group discussion.

#### 2.5.1 Data analysis

To ensure accuracy and reliability of the data, we followed a structured data cleaning and analysis process. We used a thematic analysis approach to analyze the data. First, we transcribed the audios and reviewed transcripts against audio recordings, making necessary corrections for clarity and accuracy. We then anonymized all identifying information to protect participant confidentiality. Incomplete or unclear responses were flagged for review, and redundant information was removed to focus on meaningful insights. Following data cleaning, we then constructed the themes by identifying key patterns from the data using Braun and Clarke's 6-step thematic analysis (44). First, we familiarized ourselves with the data by reading the transcripts multiple times, gaining a comprehensive understanding of the participants' experiences and perspectives. Next, we generated initial codes by manually identifying significant statements and phrases relevant to the objective of the study. We then grouped related codes into broader categories, forming the basis for themes that reflect recurring topics across the data. The categorized codes were then reviewed and refined to develop overarching themes. After developing the initial themes, we refined them by reviewing the data again, ensuring the themes were distinct, coherent, and representative of participants' responses. We also identified key patterns by examining the frequency of themes and their relationships, as well as variations based on participant demographics. Finally, we contextualized these themes within the existing literature on trauma-informed care and digital health technologies, connecting our findings to broader research. We maintained analytical rigour through reflexivity, debrief sessions, and noting of analytic decisions. We took note of our biases as indicated under researcher positionality and reflexibility, whilst recording the progression from codes to final themes. Consistent with a trauma-informed lens, the analysis emphasized sensitivity to participants' emotional safety and meaning-making, ensuring that interpretations reflected participants' experiences in their own terms. Please see Table 1 for a detailed analysis process.

**Table 1:** Data analysis process.

Examples of initial codes	Categorized codes	Overarching themes
• User-friendly interface • Some technology platforms accessed on specific devices • Lack of universal design standards	Device compatibility and user-experience	Accessibility and inclusivity
• Difficulty tolerating prolonged screen • Design options may not be sufficient • Making things a little more gender neutral • Display a pride flag on the sites	Culturally and community sensitive design	
• Multiple content formats e.g., large-print, audio • Ability to adjust text sizes • Use screen readers • Select high-contrast backgrounds	Customization and emergency	
• Waiting time can trigger trauma • Use of non-aggressive colours is essential • Include helpline • Incorporate a “panic button” feature	Privacy and safety	Safety, privacy and confidentiality
• Provide clear information about data practices • Incorporate encryption to ensure previous content can't be easily viewed • Essential to have strong security measures	Data security and practices	
• Content advisories help users navigate potentially sensitive information. • Use of expandable menus can sometimes be problematic	Navigation efficiency and information accessibility	
• Information presented in a compassionate and relatable way. • Vague terms like ‘we will get back to you shortly’ can be quite stressful. • Language used can sometimes be fear-inducing. • Use the third person for sensitive topics. • Put a clear trigger warning • Use digital flashcards	Tailored terminology	Empathetic language and terminologies
• Using inclusive language • Concerns with anatomical diagrams that are exclusive. • Present information at various levels of complexity, from basic to advanced.	Respectful framing and cultural sensitivity	
• Need for more options to talk to someone. • Put on links to crisis lines, helplines. • Add AIs if no person to speak to.	Support services and professional help	Emotional and psychological support
• Reaching isolated communities • Create platforms that link to other experts.	Connecting with community	
• Inclusive, non-graphic, non-stereotypical imager • Avoid imagery or symbols tied to specific cultural, religious, or political meanings unless intentionally inclusive	Appealing imagery	Aesthetic design
• Use red sparingly, as it can trigger associations with danger, blood, or errors • Avoid high-contrast or overly saturated colours that can feel alarming or clinical.	Calming color combination	

## 3 Findings

A total of 23 individuals participated in the focus group discussions. Out of these, 12 (52.2%) of the participants identified as women, 7 (30.4%) identified as men and 4 (13%) identified as non-binary and other gender identities. The average age was 37.2 years and ranged from 24 to 75. The first two focus groups each consisted of five participants who identified as women. The third focus group comprised five men. The fourth focus group included three non-binary participants and one woman, while the fifth focus group included two men, one woman, and one non-binary participant. All participants reported using digital health technologies to access sexual and reproductive health services, including abortion care, appointments, consultations, antenatal and postnatal care, STI testing, cancer screening, and contraception services. All the participants have indicated using web-based technologies like the provincial online STI testing platform (Getcheckedonline), and the Options for Sexual Health website to seek health services. Seven of them used video visits via Zoom for consultations, and 17 participants have visited the BC Women's Hospital website on Abortion and Contraception. While the participants may access these web-based interventions on a phone, none of them indicated using a mobile health application.

### 3.1 Thematic findings

The key findings from the focus group discussions (FGDs) are organized into five thematic areas that reflect trauma-informed approaches on how sexual and reproductive health-related digital technologies can be designed and/or used. These include (1) accessibility and inclusivity; (2) safety, privacy, and confidentiality; (3) empathetic language and terminologies; (4) emotional and psychological support; and (5) aesthetic design. The findings are presented below alongside quotes from each FDG to illustrate key points.

#### 3.1.1 Accessibility and inclusivity

To develop trauma-informed digital health technologies, the participants emphasized the need for digital health platforms to be accessible and inclusive of users with diverse identities and abilities. They specifically noted that people with disabilities may find it overwhelming to access sexual and reproductive health services from web-based platforms that are not developed with them in mind. “*For design, I think you should also pay attention to fonts […] because there are people with learning or visual disabilities who may fail to access digital services*” (FGD 3) Another participant shared that, features like screen readers are crucial to prevent re-traumatization (FGD 4), underscoring the need for inclusive design that addresses both functional and emotional needs. The participants further indicated customization as an approach to ensuring accessibility and inclusivity. For instance, they called for the need for users to adjust text sizes and select high-contrast backgrounds to enhance readability and comfort while bypassing triggering or sensitive content. One participant shared:

To create a safe and non-traumatizing environment, technology providers should ensure that their platforms offer customizable display options, such as high-contrast backgrounds like white text on black or black text on white, to enhance readability (FGD 1).

Other participants also stressed that digital health platforms should provide flexible options that allow users to tailor their experience based on personal preferences and needs, which can make the process more user-friendly and less stressful. Some participants pointed to a need for “digital flashcards” to allow technology users to choose when to read or access specific information that may be triggering. “*In terms of a design element, there are digital flashcards where you can click and flip for you to see the story […]”* (FGD 3). The participants further suggest that at the front of the flashcard, designers may add a trigger warning: “*don't click on this unless you would like to read about it*” (FGD 3). Participants from the other four FGDs also shared the same view. According to them, this not only enhances user autonomy and comfort but also gives them greater control over their experience and allows them to manage their exposure to sensitive topics.

#### 3.1.2 Safety, privacy, and confidentiality

The participants emphasized the importance of safety, privacy, and confidentiality of digital health technologies as a way of mitigating emotional trauma, particularly in the context of sexual and reproductive health services. Participants discussed the importance of incorporating emergency features, such as quick exit buttons and other discreet ways to exit or seek help when needed. One participant specifically mentioned that: “*[…] in case of any strange experience, it is important for the developers to put either a button or a function to help the user exit the tab quickly. You know, I need to feel safe before I can engage with a technology intervention* (FGD 4). Another participant from FGD 3 added that: “*incorporating a ‘panic button’ or a quick exit feature, […], is beneficial to users who might be seeking sensitive information and do not want others to see what they are looking at*”. All participants in all FGDs considered exit features very crucial for users who may feel unsafe or triggered while accessing sensitive content. Some participants suggested that these buttons should be easily accessible but also add options to clear search history (FGD 5). They also recommended including content warnings at the top pages of digital health platforms like web-based interventions to prepare users for the material they might encounter. All these suggestions are aimed at creating a safer environment for users to access SRH services.

Other participants recounted the importance of accessing sexual health services discreetly. Some SRH services like HIV prevention and treatment or abortion care are highly stigmatized, which calls for maintaining high standards of privacy and confidentiality. A participant narrated that: “*[…] there's a lot of stigma surrounding accessing SRH services. I encourage web designers to customize privacy settings to protect users from social stigma”* (FGD 2). Similarly, another participant described how privacy agreements can be confusing and inaccessible, noting that “*there's a small checkbox to agree to terms and conditions, which then leads to a lengthy document with detailed privacy policies*” (FGD 3). Such complex documents were perceived as overwhelming and offered little clarity about how users' data are managed or protected.

Additionally, two participants shared their experiences of using an online STI testing web-based platform known as “*Get Checked Online*” and indicated that this platform provided a more private and less intimidating environment for STI testing. In addition to the need for privacy, the participants also demanded transparency in how users' data is sourced, stored, and utilized, as unclear data practices can also trigger trauma, especially for vulnerable users. One reiterated the importance of privacy and data protection and shared a positive experience of the website “Get Checked Online” by stating that.

..this website can significantly enhance the user experience by minimizing direct interactions with people, which might be more comfortable for those dealing with trauma. Additionally, I'm very passionate about data privacy. When we input personal information like our name, age, and location on various websites, we often don't know how this data is being used or shared, but this website provided us with data protection guarantees (FGD 5).

Participants argued that it's crucial for digital health technologies to provide clear information about data handling practices to build trust and ensure users feel secure. The participants also emphasized the importance of users feeling confident that their data is securely stored and protected, noting that clear, trustworthy data practices are crucial in preventing re-traumatization and fostering a safe, supportive digital health environment. One participant from FGD 1 specifically mentioned that “*web designers should incorporate strong data protection measures to safeguard users” information from being exploited or accessed without consent.”* Another participant shared that: “*the frequency of data breaches and the potential misuse of personal information are growing concerns..”* emphasizing the need to protect users' rights and privacy. Participants shared that prioritizing robust security measures and transparency can help build trust and ensure user safety.

#### 3.1.3 Empathetic language and terminologies

To mitigate technology-mediated trauma in digital health, the participants also advocated for the use of empathetic language and terminologies that respect the user's comfort and preferences. For example, a participant shared: “*I have my trust in a website that is very careful and intentional in the way they communicate to users. “Whatever happened was not your fault […]’; this is more appealing than harping on the virtues of survivors”* (FGD 4). They emphasized sensitivity to users' context and the need for trauma-informed communication, helping to create a safer and more supportive environment for accessing sensitive information. In a bid to promote trauma-informed communication, a participant stressed the importance of minimizing direct human interaction in traumatic contexts, stating, “*For digital technologies, it's valuable to have options for self-service, such as signing up or managing appointments through forms and questionnaires”* (FGD 5). Participants suggested that this approach can significantly enhance user experience, which might be more comfortable for those with prior traumatic experiences. They further noted that it improves users’ autonomy and control over their interactions with web-based platforms, which can help mitigate feelings of vulnerability.

Participants also requested the selection of language or terminologies that are non-triggering, gender-inclusive and sensitive to patients' context, especially when addressing sexual health topics that could be triggering for trauma survivors. A participant from FGD 3 highlighted that: “using inclusive language, like “patient” instead of gender-specific terms such as “woman”, “man”, is crucial”. Other participants emphasized that even when using anatomical diagrams, it's crucial to ensure that these images represent a diverse range of bodies and avoid reinforcing exclusivity. For instance, they suggested that if a website decides to use a specific body part, providing neutral and inclusive imagery that doesn't default to a particular gender or body type would be beneficial. This way, visuals were considered to support understanding of a given service without inadvertently excluding or misrepresenting different experiences. One participant highlighted the significance of using gender-neutral language, stating:

Many discussions around sexual health can quickly become heteronormative or overly gendered, which can be traumatizing for individuals with different gender identities. Instead of framing content in terms of traditional gender roles, it would be more inclusive to avoid specific terms like “when a woman experiences..” and instead use more neutral language that applies broadly to all individuals. This approach helps ensure that the discussion is relevant and respectful to everyone, regardless of their gender identity or sexual orientation (FGD 3).

#### 3.1.4 Emotional and psychological support

The participants highlighted the crucial role of integrating emotional and psychosocial support services into digital health platforms to prevent triggering emotional trauma and meet users' immediate and ongoing needs. For example, a participant mentioned that: “*I would like to be told the truth and treated as a human being and not just a person in a waiting room”* (FGD 2). Others suggested: “*you can put in links to crisis lines, and helplines*” (FGD 3). Across FGDs, participants emphasized that having access to a variety of support options is vital, especially for individuals who may feel isolated or overwhelmed by the content they encounter. Several participants advocated for the inclusion of direct links to crisis intervention services, such as hotlines, live chats, and links to trained counsellors to provide immediate assistance.

They also suggested that integrating calming techniques, including guided breathing exercises or mindfulness prompts, could help users manage their emotional responses in the moment. The idea of including web-based forums and support groups was also discussed, as participants noted that these spaces can foster a sense of connection and community, particularly for those who might be experiencing isolation (FGD 3, FGD 4). They further added that: “it would be beneficial to create platforms that link to other experts and organizations working in the same field. This would foster collaboration, share resources, and provide users with access to a wider network of specialists and support services (FGD 3). Participants suggested that these platforms should facilitate access to relevant peer support groups to allow users to share their experiences and find solidarity, which can be therapeutic (FGD 1). Some participants suggested the use of AI tools like Woebot, which they considered to have shown promise in helping users identify and address thought distortions through guided, empathetic conversations. Furthermore, placing links to support services and resources at the bottom of these pages was seen as a helpful way to guide users to further seek help if they feel overwhelmed

#### 3.1.5 Aesthetic design

Another key insight was the importance of design elements like colour and font in creating a safe and calming environment for users. Participants emphasized that the use of non-aggressive colours is essential in ensuring that users feel safe and comfortable while navigating digital health platforms. Participants emphasized the importance of carefully selecting colour schemes that align with the platform's goal of providing a safe and supportive environment. Suggestions included using calming colour tones like light blue, which were perceived as soothing and conducive to a sense of safety and trust. Beyond privacy, the participants also emphasized that it's equally important to address potential stressors, like long waiting times, which can exacerbate existing trauma. One participant explained:

Something that stands out to me is colour and font. Not having aggressive or very, very bold colours on digital health websites, I think is really helpful for me to feel calmer. I think things like bright red and bright colours can be alarming. You don't expect to be getting safe sexual information from a site that's bold. In addition, some of those colours are linked to our previous traumatic experiences; therefore, it is important for the designers to consider the issue of color they choose to use on their websites (FGD 4).

The findings also revealed the need for digital health platforms to balance aesthetic design with practical functionality, ensuring that users can navigate easily without added frustration, especially for users who may already be in a vulnerable state.

## 4 Discussion

The integration of digital health technologies into sexual and reproductive health (SRH) services has significantly improved access to healthcare services for people who may not be comfortable with or do not have access to conventional health services (45). These technologies offer opportunities for greater reach, personalized care, and user autonomy in navigating sexual and reproductive health services. Despite the usefulness of digital health technologies, they may inadvertently expose users to new forms of psychological trauma and perpetuate existing vulnerabilities (19). This study sought to explore the experiences of individuals accessing sexual and reproductive health-related digital platforms, with a focus on identifying strategies to improve these technologies to better support users' mental and emotional well-being. Specifically, the research emphasized the importance of trauma-informed design in enhancing user safety and emotional support. Drawing on our findings, this discussion highlights the opportunities and challenges presented by digital health technologies in promoting SRH outcomes while mitigating the risk of trauma exposure.

The findings of this study highlight the urgent need for digital health technologies to prioritize accessibility, inclusivity, and customizable features to minimize the risk for emotional trauma, particularly when used to deliver sensitive health services such as sexual and reproductive health. These findings align with existing research, which stresses the importance of designing technologies that accommodate a wide range of user abilities and experiences to promote equitable access to health services (46–48). The findings show that digital health technologies that lack accessibility or inclusiveness can inadvertently trigger trauma or exacerbate emotional distress. Therefore, ensuring that digital health platforms are both easy to use and inclusive is essential in mitigating potential harm and enhancing the user experience. Customization options and emergency exit buttons also emerged as critical features in creating emotionally safe and trauma-informed digital environments. Participants emphasized the importance of customizable display settings and easy-to-follow, empathetic language to create non-triggering environments, which resonates with trauma-informed care principles (49). Existing literature supports this need, suggesting that trauma-sensitive designs reduce the likelihood of re-traumatization, especially when dealing with vulnerable populations accessing sensitive content (50). The demand for emergency features, such as quick exit buttons, further illustrates the importance of ensuring user safety, particularly for individuals accessing resources in potentially unsafe or triggering environments. This reflects broader trends in digital health where anonymous use and privacy features are increasingly prioritized to enhance user trust and safety (51).

Further to customization was the need for privacy, safety, and confidentiality when engaging with digital sexual and reproductive health services, particularly in the context of trauma. Privacy and safety have been of great concern in digital health for quite some time (52). The ability to maintain discretion, as illustrated by one participant's experience with “GetCheckedOnline” website, was valued for its ability to minimize interactions that might trigger anxiety or embarrassment. Concerns regarding data breaches also emerged, with participants emphasizing the need for transparency in how personal information is handled. This mirrors the broader societal concerns over data breaches and misuse of information (53–55). However, while existing literature largely focuses on data security protocols, our findings bring to light the need for simplification and transparency in terms of service agreements. To build trust and foster emotional comfort, digital health technologies must empower users by offering clear and accessible explanations of how data will be used and by providing options for deleting personal information. While Seh et al. (54) advocate for robust security protocols, the findings from this study challenge developers to go further by considering how these protocols are communicated to users in a way that reduces anxiety and fosters trust.

Some participants posited that some digital health platforms present navigational challenges, which could get patients re-traumatized if they are not able to access the services they need. Therefore, there was a need to ensure seamless navigation of digital platforms, device compatibility, and accessibility by equity-deserving groups, particularly for individuals with disabilities. These finding aligns with earlier research that calls for responsive design; ensuring platforms are optimized for a variety of devices (smartphones, tablets, computers) to support seamless user experiences for people with diverse abilities and expertise (56). Although navigational design has been underexplored in the electronic health record (EHR) literature (57), it remains a key issue even in sexual and reproductive health (SRH) platforms, where users often struggle to locate crucial information. Participants in this study expressed frustration with features like expandable menus, which, while intended to streamline content, can cause stress when seeking urgent or sensitive information (58). For trauma survivors, such navigation challenges can intensify feelings of helplessness and frustration, potentially re-traumatizing users. This highlights the importance of designing digital health technologies that are not only aesthetically pleasing but also prioritize functional and intuitive navigation. Moreover, the need for customizable display options, such as font adjustment and screen readers, reiterates the call for universal design principles that cater to users with visual or cognitive impairments (46, 59). Providing content in multiple formats, such as large-print or audio, enhances the usability of health platforms, especially for populations that may not traditionally engage with digital services (60, 61).

Another significant aspect that emerged from the discussions was the need for cultural, community-sensitive and aesthetic design. Visual design elements, such as colour and font, also played a significant role in shaping users' sense of safety (41). While some scholars have emphasized the importance of visual design in digital public health platforms (62), specific aspects like colour and font choice have received relatively little attention. Participants in this study pointed out that certain “aggressive” colours, particularly red, could inadvertently evoke feelings of danger or alarm, creating a less supportive and more provoking environment. This suggests that digital health platforms should not only focus on functionality but also consider the psychological effects of visual design on users, particularly those with trauma histories. Careful consideration of visual design is crucial in fostering a sense of safety, especially when engaging with sensitive topics like sexual and reproductive health. Subtle design choices, such as opting for more neutral or calming colours, can enhance user comfort and contribute to a trauma-sensitive experience.

It is important to note that existing literature often overlooks the emotional impact of design choices on trauma survivors, focusing instead on technical aspects (63). Our findings, however, indicate that the aesthetics of digital health platforms are as critical as their technical performance, as they contribute to the overall user experience and sense of safety. Therefore, visual design elements should be selected carefully to promote a calming, non-threatening environment, which is essential in the context of trauma-informed care. Our findings also emphasized that user-centred, accessible design must not be compromised for visual appeal, especially in platforms addressing sensitive health topics. In high-stress situations, platforms need to ensure that essential information is easy to find and interact with, reducing the risk of users feeling overwhelmed or further traumatized.

Trauma-informed communication emerged as another key area, with participants calling for empathetic language, correct use of terminologies and cultural/context-specific communication strategies when engaging with people via digital health technologies. Many participants advocated for minimizing direct human interaction in traumatic contexts, emphasizing the value of self-service options such as forms and questionnaires. This aligns with trauma-informed principles that prioritize user control and choice (11, 64). However, while these features may help reduce anxiety, they must be balanced with adequate human support for users who may need more guidance or reassurance (64, 65). Our findings suggest that digital health platforms need to offer a spectrum of interaction options, allowing users to control the degree of human involvement while still providing immediate access to support if needed. This nuanced approach challenges existing frameworks that often present self-service and human interaction as mutually exclusive options. Trauma-informed communication, plus the integration of emotional and psychological support features like live chats, could help improve the health of people who face vulnerabilities in their sexual and reproductive health journeys (66).

While the convenience of such platforms was lauded, the discussion also brought attention to how design and deployment features of digital health technologies can exacerbate stress for trauma survivors. This raises important questions about whether the current design of digital platforms truly prioritizes user well-being beyond their functional capabilities. Existing evidence has stressed the importance of privacy, confidentiality and safety in digital health settings (53, 67), yet our findings indicate that privacy and safety need to be paired with emotional design considerations to prevent additional trauma triggers. This suggests that while privacy is crucial, a purely functional approach to technology does not fully address the psychological impacts that users may experience during prolonged interactions with these platforms. As such, an integrated approach that balances discretion with prompt service is essential for trauma-sensitive care.

## 5 Strengths and limitations

Despite the potential usefulness of the findings, there were some limitations that should be considered when interpreting or applying the findings. First, data collection occurred via Zoom—potentially excluding people with no access to technology who otherwise might have provided useful information. Furthermore, other participants might not have been able to fully express themselves in the virtual focus groups due to the constraints of the digital platform, technical issues, or lack of familiarity with virtual discussions (68). Also, because of our obligation to hide participants' identities from each other, we asked them to use pseudonyms and turn off their videos. While these measures ensured a degree of anonymity, it remains possible that some participants' identities could be inferred through their voices by other participants who already knew them. In addition, although experiences and management of trauma may vary across identity groups, this study did not examine identity-specific experiences or coping strategies. Future research is needed to better understand how different groups—such as Indigenous peoples, immigrants, individuals experiencing homelessness, and religious minority groups—experience and manage trauma. Finally, although we screened participants to ensure that they met the inclusion criteria, we could not ascertain whether all those who participated in the focus group discussions did indeed meet the inclusion criteria, as the screening was based on self-report.

## 6 Conclusion

The use of digital health technologies for sexual and reproductive health is on the increase. At the same time, these technologies present real risks to potential patients, particularly marginalized and equity-deserving populations who might have prior experiences of trauma. In this study, we explored patient-centred approaches that can be adopted to minimize these risks during the design and use of digital health technologies. This study revealed very useful considerations for ensuring that sexual and reproductive health-related digital technologies are equitable, safe, and empowering for all users, particularly trauma survivors. These recommendations have been summarized in Table 2 and mapped onto the five principles of Fallot and Harris trauma-informed care (69). By integrating these principles, developers and healthcare providers can create technology applications that reduce barriers, mitigate re-traumatization risks, and promote positive health outcomes.

**Table 2:** Trauma-Informed Recommendations mapped onto Fallot and Harris five principles.

Trauma-Informed Care Principle	Design recommendation
Safety	• Use non-aggressive and calming colors e.g. light blue • Incorporate emergency exit buttons • Avoid pop-ups, auto-playing videos, and moving banners • Use empathetic and respectful language sensitive to users’ context • Use digital flashcards and add trigger warning at front of each flashcard • Allow anonymous use and options to clear search history • Include content warnings at the top of each page, not site-wide alerts
Trustworthiness	• Show certifications of staff involved in content development • Specify funding sources, partners and institutional affiliations • Incorporate strong data protection and management measures • Use simple or plain language to provide health information • Link information to evidence-based sources • Indicate who created and reviewed the content
Choice	• Let users decide how deeply to engage with sensitive topics • Information provided using multiple formats (text, audio, video) • Split information into small portions • Provide customizable display options (dark mode, font size) • Allow users to skip or bypass triggering sections • Offer a high-contrast background to enhance readability • Add screen readers to facilitate varying needs
Collaboration	• Engage users at different stages of designing the platform • Include web-based forums and support groups to foster connection and sense of belonging • Add some AI tools like Woebot to address thought distortions through guided conversations • Link platforms to other organizations working in the same field • Provide users with access to a wider network of specialists • Add links to crisis lines, live chats, helplines & links to trained counsellors to provide immediate assistance • Conduct follow-up focus groups to review implemented features
Empowerment	• Use inclusive, gender-affirming, and culturally sensitive language e.g. use “patient” instead of man or woman • Explicitly state that abuse is not the survivor’s fault • Have options for self-service e.g. managing appointments by self • Use neutral and inclusive imagery • Validate lived experiences without idealizing users or survivors • Platform should prioritize users’ needs over institutional priorities • Incorporate user feedback as visible design changes

## Data Availability

The datasets for this study are not publicly available because they contained information from vulnerable population groups that could be identifying. Requests to access the datasets should be directed to the corresponding author/s.
