# Supplementary material for: “I need to feel safe before I can engage”: embedding trauma-informed principles in sexual and reproductive health digital technologies
Source: Front Digit Health. 2026 Mar 5;8:1733713. doi: 10.3389/fdgth.2026.1733713 (PMC12999910; doi:10.3389/fdgth.2026.1733713)
Supplement: Supplementary file 1 [file Datasheet1.pdf]

Sample Interview Questions

Preamble: Welcome to this focus group discussion. My name is Agnes Kyamulabi, and I am here with [Name], both from UBC. Today, we aim to gather your insights on using digital platforms for sexual health services and any related traumatic experiences. Our goal is to understand the impacts of these technologies on trauma and to improve the design and delivery of these platforms to better support users' mental and emotional well-being. We want to generate people-centered recommended strategies for preventing technology-mediated trauma. Please note that your participation requires consent, and the session will be recorded to ensure accurate documentation.

Guidelines for the discussion:

- Stay for the full discussion but feel free to leave or pause if uncomfortable.
- Speak loudly and clearly.
- Speak one at a time and allow others to contribute.
- Share stories and examples without identifying people by name.
- Do not reveal private or personal information; focus on traumatic experiences with digital sexual health services.
- Respect the confidentiality of the discussion and each participant.

We will adhere to the time limitations of the discussion.

Icebreaker:

Start with an icebreaker to help participants feel comfortable and open up.

Example: Let's start by self-introductions and sharing a fun fact about ourselves or a memorable event.

Design preferences for trauma-informed digital health technologies

- What features do you believe are essential to ensure safety and privacy in digital health technologies?
- What privacy features are essential to prevent feelings of vulnerability or exposure
- How can technologies be designed to be more user friendly for people who have experiences of trauma
- How should sensitive information be presented to avoid causing distress or triggering trauma?
- Are there specific words, phrases, or images that should be avoided?

Emotional and psychological support

- What types of emotional support features (e.g., crisis support, calming techniques, chat support, peer support, health professional integration, counselling services) would help make digital health technologies less traumatizing?
- In your opinion, what could be the potential benefits of incorporating these features on digital health platforms.
- And what are the downsides to that

Trust

- What factors can help build your trust in using digital health technologies
- How can technology providers demonstrate their commitment to creating a safe and non-traumatizing environment?

Ideal features and participants' role

- If you could design a digital health technology to be as non-traumatizing as possible, what would it look like?
- How would you like digital technologies to function to ensure they are supportive rather than triggering?
- Imagine you are invited to take part in developing a website for a sensitive sexual health topic, what role can you play in ensuring that such a platform is non-triggering and trauma informed.
- How do you envision the future of trauma-informed digital health technologies evolving, and what advancements or improvements do you hope to see in this field?

Closing Remarks

Can you please share any final thoughts, concerns, or insights that you haven't shared yet. (Reiterate the importance of their input in shaping future developments in sexual health digital platforms). Finally, express gratitude for their participation.
